# Melatonin as a potential anticarcinogen for non-small-cell lung cancer

**DOI:** 10.18632/oncotarget.8776

**Published:** 2016-04-18

**Authors:** Zhiqiang Ma, Yang Yang, Chongxi Fan, Jing Han, Dongjin Wang, Shouyin Di, Wei Hu, Dong Liu, Xiaofei Li, Russel J. Reiter, Xiaolong Yan

**Affiliations:** ^1^ Department of Thoracic Surgery, Tangdu Hospital, The Fourth Military Medical University, Xi'an, China; ^2^ Department of Thoracic and Cardiovascular Surgery, Affiliated Drum Tower Hospital of Nanjing University Medical School, Nanjing, Jiangsu, China; ^3^ Department of Biomedical Engineering, The Fourth Military Medical University, Xi'an, China; ^4^ Department of Ophthalmology, Tangdu Hospital, The Fourth Military Medical University, Xi'an, China; ^5^ State Key Laboratory of Cardiovascular Disease, Fuwai Hospital, National Center for Cardiovascular Diseases, Chinese Academy of Medical Sciences & Peking Union Medical College, Beijing, China; ^6^ Department of Cellular and Structural Biology, UT Health Science Center, San Antonio, TX, USA

**Keywords:** melatonin, non-small-cell lung cancer, oncostatic effects, drug synergy, potential directions

## Abstract

Non-small-cell lung cancer (NSCLC) is a leading cause of death from cancer worldwide. Melatonin, an indoleamine discovered in the pineal gland, exerts pleiotropic anticancer effects against a variety of cancer types. In particular, melatonin may be an important anticancer drug in the treatment of NSCLC. Herein, we review the correlation between the disruption of the melatonin rhythm and NSCLC incidence; we also evaluate the evidence related to the effects of melatonin in inhibiting lung carcinogenesis. Special focus is placed on the oncostatic effects of melatonin, including anti-proliferation, induction of apoptosis, inhibition of invasion and metastasis, and enhancement of immunomodulation. We suggest the drug synergy of melatonin with radio- or chemotherapy for NSCLC could prove to be useful. Taken together, the information complied herein may serve as a comprehensive reference for the anticancer mechanisms of melatonin against NSCLC, and may be helpful for the design of future experimental research and for advancing melatonin as a therapeutic agent for NSCLC.

## INTRODUCTION

Lung cancer, which accounts for about 13% of total cancer diagnoses, is the most frequently diagnosed cancer and the leading cause of cancer death among males and females [1]. More than 85% of lung cancer cases are classified as non-small-cell lung cancer (NSCLC) [2], of which the predicted 5-year survival rate is only 15.9% [3]. Surgery and chemoradiotherapy are the two major treatments to prolong the survival of NSCLC patients, but improvements are marginally effective [4]. Moreover, radio- or chemotherapies often lead to undesirable side effects on normal cells or tissues, which limits their use as a treatment for cancer [5]. Thus, a number of studies were devoted to overcoming the deleterious effects and enhancing the efficacy of these treatments when given in combination with the appropriate supplement such as melatonin, which is well known lack of toxic and undesirable side effects [5–8].

Melatonin (N-acetyl-5-methoxytryptamine) is an indoleamine produced by the pineal gland that modulates the human circadian rhythms and acts as a neuromodulator, cytokine, biological response modifier and antioxidant [9, 10]. As a potent free radical scavenger and antioxidant, melatonin has the capacity to scavenge up to 10 reactive oxygen species (ROS) *via* the cascade reaction means, which distinguishes melatonin from the classic antioxidants that scavenge only one or less ROS [11–13]. Additionally, numerous publications have reported that melatonin inhibits the growth of a variety of cancers: lung [5, 14, 15], breast [16–20], prostate [21–24], liver [25, 26], colon [27, 28], etc. The oncostatic mechanisms of melatonin are related to several hallmarks of cancer, including anti-proliferation [14], induction of apoptosis [5, 14, 15], inhibition of invasion and metastasis [29, 30], anti-angiogenesis [16, 31], and enhancement of immunomodulation [32] among others. Furthermore, clinical studies have demonstrated that melatonin treatment enhances the efficacy and reduces the side-effects of chemotherapy, prolongs survival time, and improves quality of life for NSCLC patients [8, 33]. Accordingly, these findings attest to melatonin being a potential anticancer drug in the treatment of NSCLC and provide an inducement for further work in this area.

In this review, we focus on the therapeutic actions of melatonin as a treatment for NSCLC. First, consider the correlation between melatonin disruption and its impact on NSCLC incidence, and the anticarcinogenic effects of melatonin against lung cancer. We then describe in-depth the oncostatic effects of melatonin and the drug synergy of melatonin for NSCLC treatments. Finally, we discuss several novel potential directions for future research in this area. The information complied herein may serve as a comprehensive reference for the anticancer mechanisms of melatonin against NSCLC, and may be helpful for the design of future experimental research and for advancing melatonin as a therapeutic agent for NSCLC.

## MELATONIN DISRUPTION AND NSCLC

**Figure 1:**
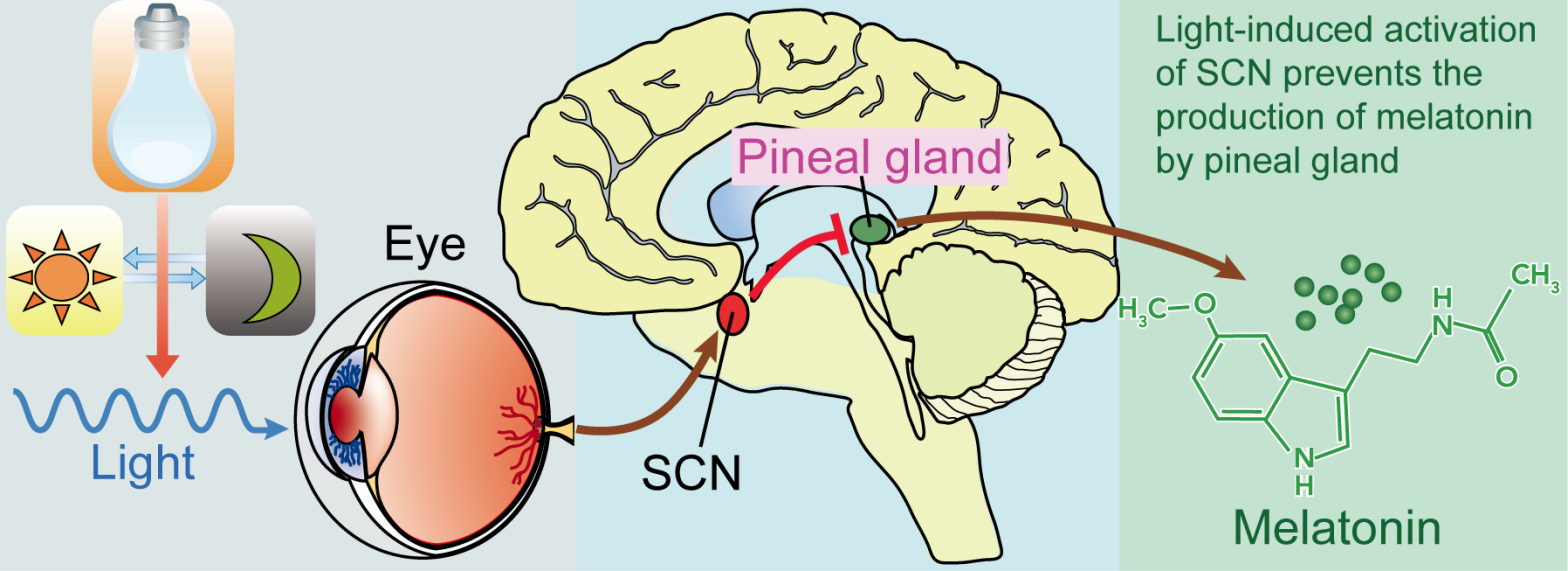
Light, suprachiasmatic nuclei (SCN), and the pineal/melatonin circuit Melanopsin in retinal ganglion cells in the eye respond to light (natural or artificially) and transmit signals to the SCN. Then light-induced activation of the SCN prevents the pineal gland from producing melatonin and; conversely, melatonin production and secretion is increased during the dark period.

Melatonin, the “chemical expression of darkness” [34], is an important component of the body's internal time-keeping system [35]. However, light exposure by artificial illumination at night suppresses human melatonin levels and disrupts circadian rhythmicity (mechanisms are illustrated in Figure 1) [36, 37]. Under controlled laboratory conditions, retinal exposure to illuminances as low as 1 lux or less of monochromatic light at wavelengths of 440 to 460 (blue-appearing light) significantly lowers nocturnal melatonin levels [38–40], as does < 100 lux of broad spectrum fluorescent light [41–44]. Disturbances in melatonin circadian rhythm result in chronodisruption which is associated with many health disorders including heart diseases, neurodegenerative diseases, and cancer [11, 45]. Epidemiological studies have demonstrated that melatonin disruption would increase the risk of cancers, including breast cancer [46–48], prostate cancer [49, 50], and colon cancer [50, 51], etc. Additionally, melatonin disruption also can increase the risk of NSCLC (Table 1). Parent *et al.* [50] reported that men who ever worked at night anytime as an adult have higher risk for lung cancer, with excesses apparent across all main histologic subtypes. Advanced NSCLC patients suffered from poor sleep quality and disrupted circadian function [52]. Data have revealed that melatonin levels are lower in NSCLC patients regardless of TNM stages [53]. Moreover, Hu *et al.* [54] demonstrated that melatonin and its metabolite, 6-sulfatoxymelatonin, are significantly lower in NSCLC patients with standard chemotherapy.

**Table 1:** Melatonin disruption and NSCLC

Research object	Measures	Outcome	References
Epidemiologic surveys			
761 male lung cancers (142 small-cell carcinomas, 149 adenocarcinomas, 314 squamous cell carcinoma and 156 others) and 512 controls in Montreal	Face to Face interviews	Compared with men who never worked at night, the adjusted OR among men who ever worked at night was 1.76 (95% CI: 1.25, 2.47) for lung cancer. According to main histologic subtype, adjusted ORs were 1.91 (95% CI: 1.27, 2.87) for squamous cell carcinoma, 1.62 (95% CI: 1.25, 2.47) for small-cell carcinoma, and 1.46 (95% CI: 0.86, 2.50) for adenocarcinoma	Parent *et al*. [50]
Experimental studies			
Female BD2F1 mice with subcutaneous propagation of Lewis lung carcinoma	Tumor inoculation and melatonin treatment (1.25 mg/kg/night) were performed 2 months after pinealectomy	Melatonin treatment decreased metastasis with consequent restoration of thymic efficiency, negative crude zinc balance and IL-2 production in compare with controls	Mocchegiani *et al*. [32]
Melatonin level in lung cancer patients			
30 NSCLC patients before and after treatment with standard chemotherapy (cisplatin plus vinorelbine) and 63 healthy volunteers	Blood samples were collected at 12 noon and 12 midnight. Urine samples were collected at 7 AM and 4 PM	Melatonin, its precursor tryptophan, and its major metabolite, 6-sulfatoxymelatonin concentrations were significantly lower in cancer patients, in comparison with healthy subjects. Furthermore, those concentrations progressively decreased after standard chemotherapy in NSCLC patients	Hu *et al*. [54]
17 patients with stages I and II of untreated NSCLC, 17 patients with stages III and IV of untreated NSCLC, and 17 controls	Melatonin serum level was measured in blood samples collected every four hours for 24 hours	Melatonin levels were lower in the patients with NSCLC than in normal subjects, without a significant difference between the two groups of cancers, but a clear circadian rhythm was present in the three groups	Mazzoccoli *et al*. [53]

Collectively, the published literature suggested that melatonin disruption increases the risk of NSCLC. The mechanism involved, however, warrant further investigation. It is possible that melatonin anticarcinogenic actions may account for the increased incidence of NSCLC. This will be discussed later in this review.

## EFFECT OF MELATONIN ON LUNG CARCINOGENESIS

Melatonin is an experimentally documented anticarcinogen against a number of cancers [55]. Previous animal studies have demonstrated that melatonin inhibits different chemical mutagen-induced carcinogenesis of breast [56–58], liver [59], colon [60, 61], uterine cervix and vagina [62]. Moreover, melatonin limits the lung carcinogenesis induced by urethane in A/J and SHR/u mice. When compared with the urethane-treated animals, melatonin treatment also significantly lowered concentration of serum malondialdehyde (MDA), an index of the lipid peroxidation (LPO), this indicated a potential role of melatonin as an antioxidant in relation to its cancer inhibiting actions [55, 63].

**Figure 2:**
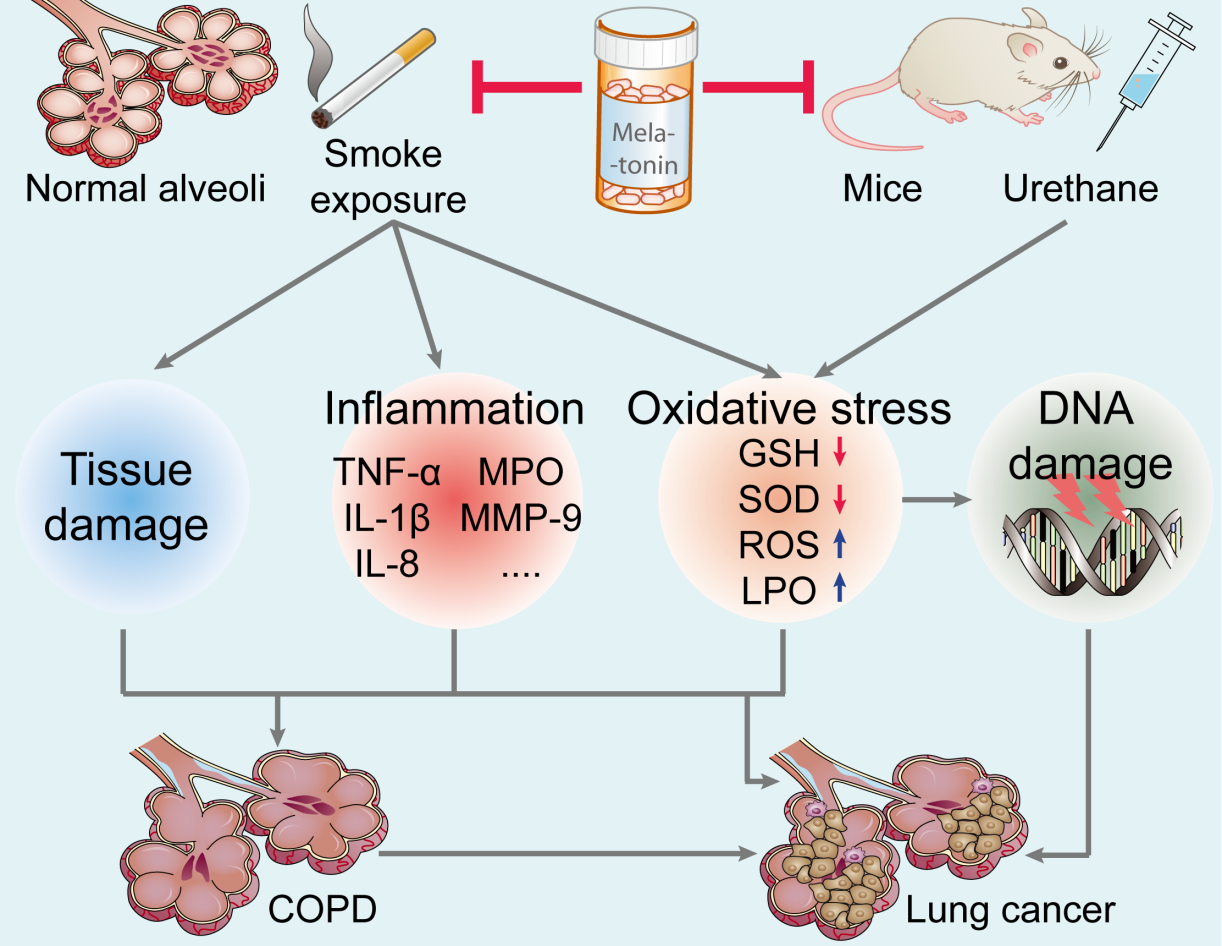
Effect of melatonin on the prevention of lung carcinogenesis Melatonin inhibits urethane-induced lung carcinogenesis in mice. Moreover, melatonin attenuates cigarette smoke-induced lung tissue damage, inflammation, and oxidative stress. Melatonin may reduce the incidence of lung cancer and lung diseases (such as COPD, a key risk factor for lung cancer). TNF-α, tumor necrosis factor-α; IL-1β, interleukin-1β; MPO, myeloperoxidase; MMP-9, matrix metalloproteinase-9; GSH, glutathione; SOD, superoxide dismutase; ROS, reactive oxygen species; LPO, lipid peroxidation; COPD, chronic obstructive pulmonary diseases.

Cigarette smoke exposure, both active and passive, is a major epidemiologically proven cause of lung cancer; this accounts for about 90% of lung cancer incidence [64]. In the gaseous and particulate phases, cigarette smoke contains more than 4,500 components, including direct carcinogens (e.g., methylcholanthrene, benzo-α-pyrenes and acrolein), toxins (e.g., carbon monoxide and nicotine), reactive solids with chemically catalytic surfaces, and oxidants (e.g., superoxide and nitrogen oxides) [65, 66]. Additionally, cigarette smoke is also considered as a critically-important risk factor in the development of chronic obstructive pulmonary diseases (COPD), which is another key risk factor for lung cancer [64, 66]. Active smoking decreases human blood melatonin levels [67], while melatonin treatment attenuates cigarette smoke-induced pulmonary diseases and lung cancer mainly by three potential mechanisms (see Figure 2). First, melatonin may be useful in reducing lung tissue injury caused by smoke-related toxins (e.g., nicotine) [68, 69]. Furthermore, melatonin may decrease pulmonary inflammation induced as a consequence of smoking [70]. In cigarette smoke exposed mice, Shin *et al.* [71] reported that melatonin significantly reduced neutrophils counts, suppressed proinflammatory cytokines (TNF-α, IL-1β, IL-8, IL-6), and decreased matrix metalloproteinase (MMP)-9 and myeloperoxidase (MPO) expression in lung tissue. As an effective antioxidant, melatonin inhibition of oxidative stress induced by smoke is a potentially important protective means by which it reduces lung damage. In the lung tissue of smoke-treated animals, melatonin significantly reduced reactive oxygen species (ROS) and LPO production, and upregulated glutathione (GSH) and superoxide dismutase (SOD) activity [68, 71]. Thus, the likelihood is high that melatonin reduces respiratory DNA damage caused by ROS, thereby inhibiting carcinogenesis [72]. The collective evidence suggests that melatonin should be considered a worthy agent for inhibition of lung cancer.

## EFFECT OF MELATONIN ON NSCLC CELL APOPTOSIS

**Figure 3:**
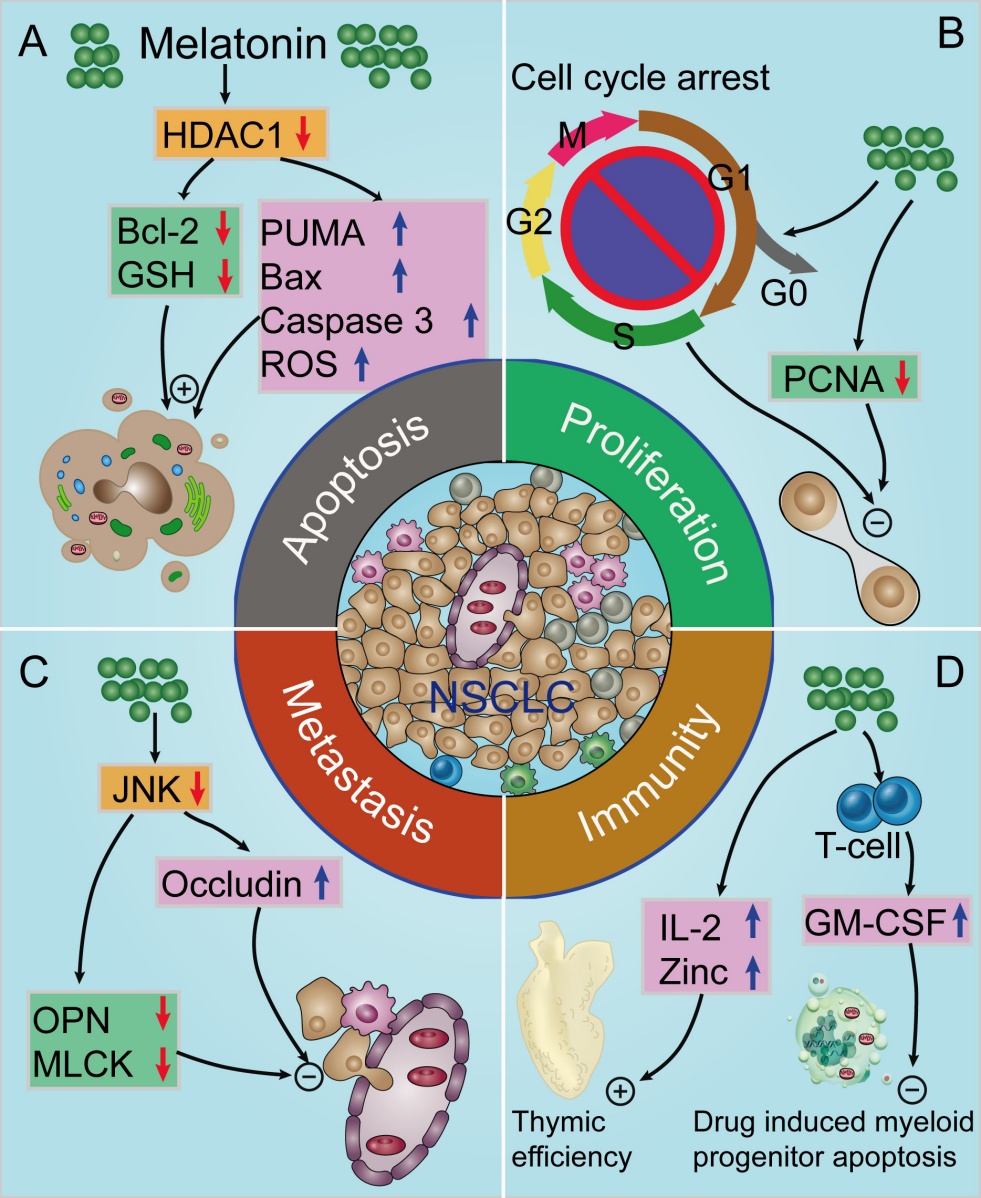
Proposed oncostatic actions of melatonin on the hallmarks of NSCLC **A.** Melatonin treatment induces NSCLC cells apoptosis; **B.** melatonin inhibits NSCLC cell proliferation; **C.** melatonin supplementation suppresses NSCLC cells metastasis; **D.** melatonin has indirect anti-cancer effects *via* enhancement of immunomodulatory activity. HDAC1, histone deacetylase-1; Bcl-2, B-cell lymphoma-2; GSH, glutathione; PUMA, p53 up-regulated modulator of apoptosis; Bax, Bcl-2 associated X protein; ROS, reactive oxygen species; PCNA, proliferating-cell nuclear antigen; OPN, osteopontin; MLCK, myosin light chain kinase; IL-2, interleukin-2; GM-CSF, granulocyte-macrophage colony-stimulating factor.

Resisting cell death is one of the hallmarks of cancer [73]. Apoptosis is a well-known mechanism of programmed cell death that does not injure normal neighboring cells and reduces local inflammation [74]. Since programmed cell death by apoptosis serves as a natural barrier to cancer development [73], apoptotic induction is an important mechanism initiated by chemotherapeutic agents [5]. Melatonin exerts anti-apoptotic effects in normal cells exposed to toxic agents or metabolic injury. However, melatonin usually has the opposite effect on cancer cells and induces apoptosis in a wide range of different tumors (e.g., breast, prostate, cervix, liver, colon, pancreas, kidney, neuro) [18, 20, 24, 25, 28, 75–78]; thus, the induction of apoptosis of cancer cells (but not of normal cells) is one of the mechanisms by which melatonin limits tumor growth [61, 79–88]. This specifically relates to lung cancer given that melatonin treatment dose- and time- dependent decreased the viability of human A549 and PC9 lung adenocarcinoma cells, and increased their apoptotic index. Furthermore, melatonin treatment significantly upregulated the expression of Bcl-2 associated X protein (Bax), p53 upregulated modulator of apoptosis (PUMA) and ROS, enhanced the caspase 3 activity, and downregulated the B-cell lymphoma-2 (Bcl-2) and GSH levels *via* the inhibition of histone deacetylase-1 (HDAC1) signaling pathway in adenocarcinoma cells [14] (Figure 3A). Moreover, Plaimee *et al.* [15, 30] have demonstrated that melatonin induces apoptosis in SK-LU-1 human lung adenocarcinoma cells. In combination with cisplatin, melatonin can potentiates cisplatin-induced apoptosis and cell cycle arrest in the S phase [5]. Thus, it is evident that melatonin can induce NSCLC cell apoptosis, making it potentially useful as an NSCLC treatment especially in combination with chemotherapeutic agents [74, 75].

## EFFECT OF MELATONIN ON NSCLC CELL PROLIFERATION

Arguably the most fundamental trait of cancer cells involves their ability to sustain chronic proliferation [73]. Previous studies have reported that melatonin suppresses the proliferation of several cancer cells and can be viewed as an anti-mitogen [14, 89, 90]. Proliferating-cell nuclear antigen (PCNA) is a molecular marker for proliferation because of its role in cell division; the inhibition of PCNA is considered to be a viable anticancer strategy [91]. Our recent study demonstrated that melatonin supplementation downregulated PCNA expression and reduced the viability in both lung cancer A549 and PC9 cells [14]. In SK-LU-1 cells, melatonin treatment reduced the intensity of nucleic acid/DNA [15]. When SK-LU-1 cells were co-cultured with human peripheral blood mononuclear cells (PBMC), it induced the cell cycle arrest in the G0/G1 phase; this may be also related to the enhancement of immunomodulatory effect of melatonin [30] (Figure 3B). Considering the data discussed, it is likely that the anti-proliferative actions of melatonin may be related to its anticancer activity.

## EFFECT OF MELATONIN ON NSCLC CELL METASTASIS

Greater than 90% of cancer-related mortalities result from metastases [92, 93]. Lung cancer cells metastasize to brain, bone, contra-lateral lung, liver and kidney, and metastatic growths are major cause of cancer-related mortality in men and women worldwide [94, 95]. Metastatic lesions are manifested in multiple steps, including localized invasion and intravasation at the primary tumor site, sustained survival in circulation, extravasation at distant organ site and colonization at the new site [93, 96]. Several reports have suggested that melatonin inhibits NSCLC metastasis. In the pinealectomized mice bearing Lewis lung carcinoma, melatonin treatment leads to significant decrement of in metastatic volume [32]. Furthermore, Zhou *et al.* [29] reported that Melatonin significantly inhibits the migration of A549 cells; this may be associated with the down-regulation of the expression of osteopontin (OPN), myosin light chain kinase (MLCK), phosphorylation of myosin light chain (MLC), and up-regulation of the expression of occludin *via* the inhibition of c-jun-N-terminal kinases (JNK) signaling pathway (Figure 3C). Accordingly, melatonin has the potential to reduce the malignancy of NSCLC through the inhibition of cancer cells metastasis and progression. This would be consistent with a large number of reports showing that melatonin inhibits the epithelial-to-mesenchymal transition and metastases of a variety of cancer cell types [17, 97, 98]. Rho-associated kinase and its isoforms (ROCK-1 and ROCK-2) promote cancer invasion and migration by regulating actin rearrangements in the cytoskeleton [99]. A recent study by Borin *et al.* [17] reported that melatonin was effective in controlling metastatic breast cancer *in vitro* and *in vivo*, not only *via* suppression of the proliferation of cancer cells but also through suppression of cancer metastasis by ROCK-1 inhibition. Whether melatonin treatment inhibits NSCLC metastasis through antagonism of the ROCK pathway warrants further investigation.

## EFFECT OF MELATONIN ON IMMUNOMODULATION OF NSCLC

Correlation of immune system with cancer is a dynamic and complex process, which involves their reciprocally modulation of each other [61, 100]. The major component of anticancer immunity involves of both adaptive and innate immune responses, including the involvement of tumor antigen-specific cytotoxic T (CTL) and T effector (T_eff_) cells, B cells, macrophages, natural killer (NK) cells, and NK-T cells, etc. [100, 101]. Accordingly, strengthening anticancer immunity is considered an effective means of promoting cancer regression [102, 103].

Melatonin plays an important role in the immune system [104, 105]. Circulating melatonin decreases with age coinciding with the age-related decline of immune system, which is referred to as immunosenescence, contributing to an increased susceptibility to infectious diseases, autoimmunity and cancer [106, 107]. Supplementation with melatonin prevents or delays the functional deterioration of immune system during aging [107]. Melatonin treatment prevents glucocorticoid-induced [108, 109] or age-related thymocytes apoptosis [110, 111]; also, melatonin restores the degenerated thymus by increasing thymus weight and the total number of thymocytes [110, 112]. Moreover, melatonin treatment also stimulates the production of natural killer (NK) cells and macrophage/monocyte lineage cells in both the marrow and the spleen [107, 113–115]. The elevated NK cell number and function induced by melatonin are attributed partly to the cytokines produced by melatonin-stimulated T-helper cells, including IL-2, IL-6, IL-12, and IFN-γ [105, 115].

Mounting evidence indicates that melatonin modulates cancer immunity to inhibit the development of NSCLC. Melatonin induced cytokine production has been shown in human PBMC for IL-1, IL-2, IL-6, IL-12 and IFN-γ [116–119]. When SK-LU-1 cells were co-cultured with PBMC, Plaimee and colleagues [30] reported that an indirect effect is exhibited at lower doses of melatonin, enhancing human PBMC to counteract proliferation, increase apoptosis and oxidative stress in cancer cells. Moreover, Mocchegiani *et al.* [32] observed that melatonin due to its immunoenhancing properties decreased Lewis lung carcinoma metastasis in mice, with consequent restoration of thymic efficiency and increment of IL-2 production. Furthermore, in Lewis lung carcinoma bearing mice, melatonin rescued myeloid progenitor cells from chemotherapy induced apoptosis *via* a mechanism involving the endogenous production of granulocyte-macrophage colony-stimulating factor (GM-CSF), which is produced by melatonin-stimulated bone marrow T cells [114] (Figure 3D). Collectively, these results imply that melatonin may have an indirect anticancer effect on NSCLC cells by enhancing of immunomodulation.

## DRUG SYNERGY OF MELATONIN WITH CHEMOTHERAPY

Melatonin is an endogenous substance with low toxicity and favorable compatibility [61]. In human volunteers, oral administration of melatonin in doses of 1-300 mg or 1 gram of melatonin daily for 30 days resulted in no observable negative side effects [120, 121]. Combination studies of melatonin and chemotherapy have been reported to enhance the efficacy of drug against NSCLC cells and reduce the adverse therapeutic side effects [5, 30, 122, 123] (see Table 2). In experimental studies, Kontek *et al.* [122] observed that the combination of melatonin and irinotecan increases the amount of DNA damage in A549 cells, but was not effective in inducing DNA damage in healthy human lymphocytes. Furthermore, melatonin supplementation rescued myeloid progenitor cells from chemotherapy-induced apoptosis [114]. Moreover, melatonin plus cisplatin or doxorubicin enhanced the cytotoxicity of chemotherapy against lung adenocarcinoma cells [5, 124]. Berberine, a plant-derived agent, also exhibits anticancer effects. Lu *et al.* [125] reported that treatment with melatonin effectively increased berberine-induced apoptosis, and enhanced the berberine-mediated inhibition of cell proliferation, colony formation and cell migration in H1299 and A549 cells.

**Table 2:** The drug synergy of melatonin in NSCLC

Cancer categories	Number of patients	Drugs and dose	Outcome	References
Experimental studies				
SK-LU-1 NSCLC cell line	None	Melatonin (1, 2 mM) + cisplatin (10-200 μM) (48 h in culture)	In the drug combination, 1 and 2 mM melatonin reduced IC_50_ concentration of cisplatin alone from 50 μM to 11 and 4 μM. Thus, melatonin enhances cisplatin-induced cytotoxicity and apoptosis in SK-LU-1 cells and induces cell cycle arrest in the S phase in contrast to cisplatin alone group	Plaimee *et al*. [5]
A549 cells and healthy human lymphocytes	None	Melatonin (50 μM) + irinotecan (7.5, 15, 30, and 60 μM)	The combination treatment resulted in an increase in the amount of DNA damage in A549 cells, but was not effective in inducing DNA damage in healthy human lymphocytes	Kontek *et al*. [122]
A549 cells	None	Melatonin (0.1, 1 mM) + doxorubicin (0.1, 1 microg/ml)	Melatonin intensified cytotoxicity of doxorubicin in all cell lines, significantly decreasing cell numbers and promoting apoptosis	Fic *et al*. [124]
Female C57B/6 mice with subcutaneous propagation of Lewis lung carcinoma	None	Melatonin (1 mg/kg) + cyclophosphamide (40, 160 mg/kg) + etoposide (20, 40 mg/kg)	Melatonin can rescue myeloid progenitor cells from chemotherapy-induced apoptosis via a mechanism involving the endogenous production of GM-CSF by T cells	Maestroni *et al*. [114]
H1299 and A549 cells	None	Melatonin (1 mM) + berberine (20μM to 200 μM)	Melatonin sensitized NSCLC cells to berberine and enhanced the growth inhibitory effect of berberine by activating caspase/Cyto C and inhibiting AP-2β/hTERT, NF-κB/COX-2 and Akt/ERK signaling pathways	Lu *et al.* [125]
Clinical trials				
Untreatable metastatic NSCLC or GI cancers	846	Melatonin (20 mg/day) + IL-2 (3 million IU/day, 5 days/week, 4 weeks) + supportive care	The combination treatment provided a further improvement in the percentage of tumor regressions and of 3-year survival with respect to melatonin or supportive care alone	Lissoni *et al*. [127]
Advanced lung adenocarcinoma	23	Melatonin (20 mg/day) + somatostatin (1-3 mg/day) + Retinoids (5 ml) + Vitamin D (0.3 mg/day) + bromocriptine (2.5 mg/day) + cyclophosphamide (150 mg/day)	Patients with combination treatment had a median overall survival of 95 days, with very modest toxic effects and an improvement in both respiratory and general symptoms associated with length of survival	Norsa *et al*. [123]
Untreated metastatic NSCLC	147	Melatonin (20 mg/day) + cisplatin plus etoposide or gemcitabine	The 2-year survival rate and the overall tumor regression rate achieved in patients concomitantly treated with melatonin was significantly higher than that found in those treated with chemotherapy alone	Lissoni *et al*. [33]
Untreated metastatic NSCLC	100	Melatonin (20 mg/day) + cisplatin (20 mg/m2/day) + etoposide (100 mg/m2/day)	Overall tumor regression rate and the 5-year survival results (49%) were significantly higher in patients concomitantly treated with melatonin. In particular, no patient treated with chemotherapy alone was alive after 2 years	Lissoni *et al*. [8]
Advanced NSCLC	70	Melatonin (20 mg/day) + cisplatin (20 mg/m2/day) + etoposide (100 mg/m2/day)	The percent of 1-year survival was significantly higher in patients treated with melatonin plus chemotherapy than in those who received chemotherapy alone (15/34 vs. 7/36, P <0.05)	Lissoni *et al.* [129]

GM-CSF, granulocyte-macrophage colony-stimulating factor; Cyto C, cytochrome C; AP-2β, activator protein 2β; hTERT, telomerase reverses transcriptase; NF-κB, nuclear factor κB; COX-2, cyclooxygenase 2; ERK, extracellular signal-regulated kinase

**Figure 4:**
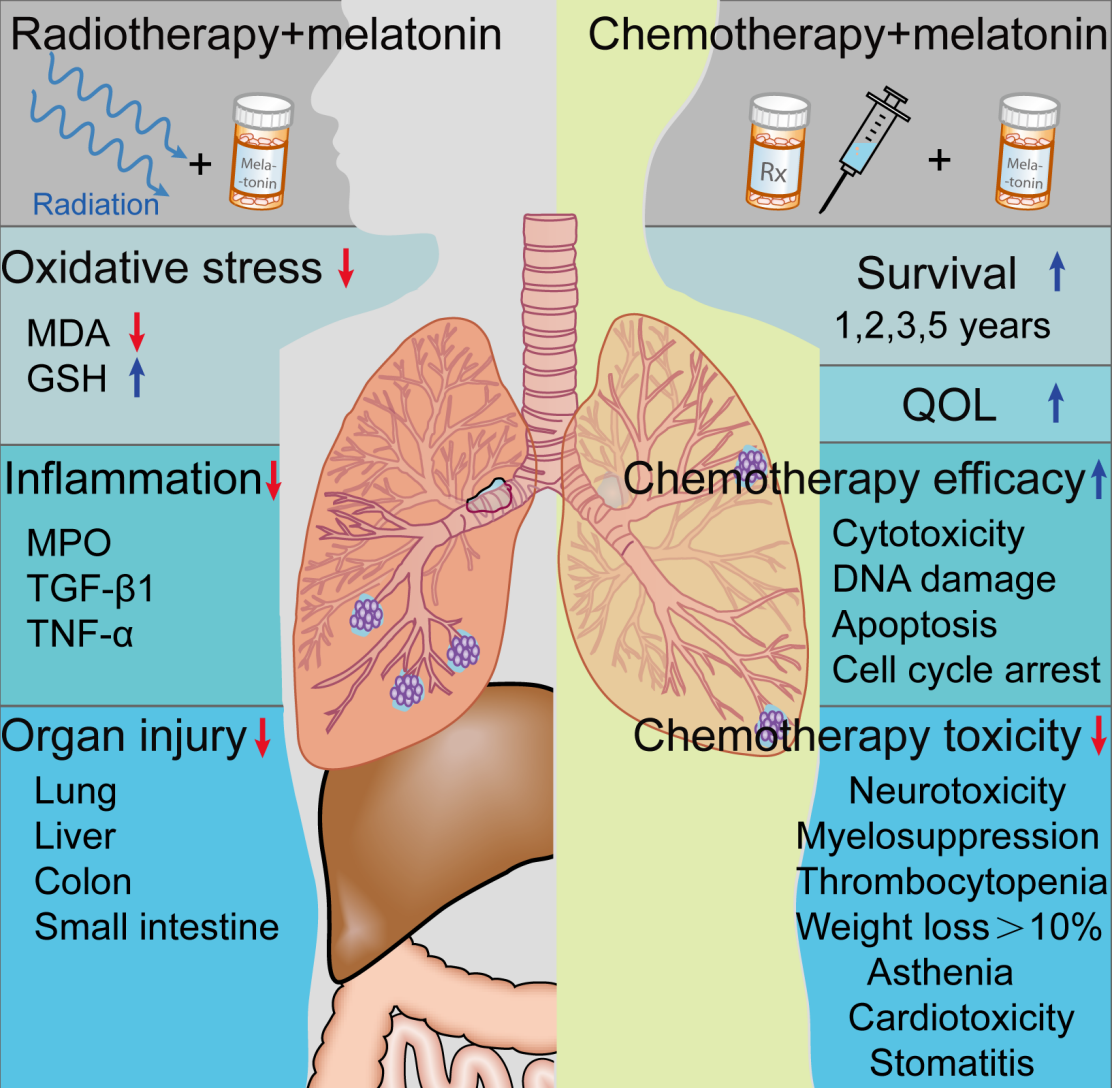
Effect of melatonin on radio- or chemotherapy Melatonin treatment reduces radiation-induced lung injury in animal studies. Moreover, melatonin supplementation may significantly promotes the efficacy of chemotherapy, reduces chemotherapy toxicity, and improves the survival and quality of life (QOL) of NSCLC patients. MPO, myeloperoxidase; MDA, malondialdehyde; TGF-β1, transforming growth factor-β1; TNF-α, tumor necrosis factor-α; GSH, glutathione.

The existence of cancer-related immunosuppression has been demonstrated in several experimental and clinical observation [126, 127]. Melatonin stimulated IL-2 production *via* activating specific melatonin receptors expressed by TH1-lymphocytes [106]. In small clinical trials, Lissoni *et al.* [127] reported that the combination treatment of melatonin with IL-2 further promotes tumor regressions and improve 3-year survival in advanced NSCLC patients. Moreover, melatonin significantly enhanced the efficacy of the standard anticancer chemotherapies (cisplatin and etoposide), and improved the 1-, 2-, and 5-year survival in untreated metastatic NSCLC patients [8, 33, 128, 129]. Additionally, melatonin supplementation also improved quality of life (QOL) in patients suffering with NSCLC [130], and reduced chemotherapy related toxicity, including neurotoxicity, thrombocytopenia, myelosuppression, asthenia, cardiotoxicity, stomatitis, and weight loss by greater than 10%; however, melatonin did not limit alopecia or anemia [8, 33, 128, 129, 131] (Figure 4). However, Egidio *et al.* [132] suggested that 20 mg melatonin given orally at night did not improve appetite, weight, or QOL compared with placebo after studying 48 patients with advanced lung or gastrointestinal cancer. Although the outcome of current studies are inconsistent relative to the efficacy of melatonin treatment for advanced NSCLC, a large scale NSCLC patient sample given melatonin could yield important findings.

Although the use of EGFR tyrosine kinase inhibitors (TKIs), such as gefitinib and erlotinib, to treat advanced NSCLC patients has become a standard of care, the use of TKIs in NSCLC patients with T790M EGFR mutations causes drug resistance [133, 134]. The most common mechanism to explain the resistance seen in EGFR mutation patients is the development of a secondary point mutation in the EGFR active domain, substituting a bulky methionine amino acid for threonine (T790M) [135]. Currently, the T790M mutation is estimated to represent 50-60% of resistance to the first and second generation EGFR mutation TKIs [136]. Recent work by Yun *et al.* [133] reported that co-treatment of gefitinib with melatonin effectively decreased the viability, downregulated EGFR phosphorylation, and induced apoptosis in H1975 cells with T790M somatic mutation. These findings indicate that melatonin plus gefitinib may be an effective treatment for NSCLC with EGFR mutations resistant to TKI-monotherapy; clinical trials should be carried out to test these combination therapy. Moreover, whether melatonin enhances the efficacy of targeted therapy in NSCLC also warrants further investigation.

## MELATONIN AND RADIOTHERAPY

Conventionally, fractionated radiotherapy for NSCLC consists of 1.8-2.0 Gy fractions given once daily for 5 days each week for a total dose of 60 Gy or more [137]. This treatment strategy is associated with improved locoregional control and survival. This treatment, however, also causes radiation-induced lung toxicity, including pneumonitis and pulmonary fibrosis [138]. It has long been recognized that the damaging effects of ionizing radiation are brought about by both direct (DNA damage) and indirect (production of highly reactive free radicals) mechanisms [120]. In 1993, the initial evidence was provided related to the protective effects of melatonin against electromagnetic radiation by ultraviolet light [139]; thereafter, numerous papers have documented that melatonin is an effective agent against radiation-induced tissue damages [120, 140–142].

Several animal studies suggested that melatonin treatment can reduce ionizing radiation-induced lung injuries [7, 143, 144]. When the thoracic regions of rats were irradiated (18 Gy), melatonin administration inhibited lipid peroxidation and radiation-induced lung injury [144]. Furthermore, Jang *et al.* [143] reported that melatonin reduced radiation (12 Gy)-induced lung injury in mice a shown by significant reduction in oxidative stress and of the production of cytokines, including TGF-β1 and TNF-α. Moreover, Sener and colleagues [7] reported that melatonin reduced damage to the lung, liver, colon and ileum, after whole body irradiation (800 cGy) to rats (Figure 4) and Vijuyalaxmi and co-workers [145] found that melatonin preserved the survival of mice exposed to a lethal dose of ionizing radiation (815 cGy). In sum, results suggest that supplementing cancer patients with adjuvant therapy of melatonin may alleviate the symptoms due to radiation-induced organ injury. Additionally, the efficacy of melatonin in combination with radiotherapy should be tested in NSCLC patients.

## POTENTIAL DIRECTIONS

Research related to the anticancer actions of melatonin on NSCLC cells has made only minor progress. One aspect that should be further examined is the anti-angiogenic actions of melatonin in NSCLC [27, 146]. Vascular endothelial growth factor (VEGF) is a highly active angiogenic factor, and the evidence of abnormally high blood VEGF levels has been proven to be associated with poor prognosis in cancer patients [147]. Lissoni *et al.* [31] reported that melatonin treatment reduces serum VEGF levels in advanced cancer patients (containing 8 NSCLC patients). Moreover, Dai *et al.* [148] suggested that melatonin suppresses endogenous VEGF expression in A549 cells. However, the mechanisms of melatonin's antiangiogenic in any tumor including NSCLC are still unclear. Numerous studies have documented that melatonin exhibits antiangiogenic actions, in part, by downregulating VEGF and HIF-1α in several cancers *via* different mechanisms; the cancer types included breast [16, 149], prostate [150–152], colon [153, 154], liver [155], and pancreatic cancer [156, 157]. These studies may provide reference for the future work in the NSCLC research field.

Fibroblasts are a component of the tumor microenvironment. Activated fibroblasts release mediators such as growth factors, cytokines and immune modulators [158]. Fibroblasts with this altered phenotype are termed cancer-associated fibroblasts (CAFs) which largely contribute to the establishment of a reactive tumor stoma that is permissive of even conductive to cancer cell survival [158, 159]. Kim *et al.* [160] suggested that melatonin suppresses acrolein-induced IL-8 production *via* extracellular signal-regulated kinases 1/2 (ERK1/2) and phosphatidylinositol 3-kinase (PI3K)/Akt signal inhibition in human pulmonary fibroblasts (HPFs). These results imply an correlation between melatonin and HPFs, but whether melatonin reduces the secretion of potent oncogenic molecules by CAFs, such as TGF-β [161] and hepatocyte growth factor (HGF) [162], or inhibits other tumor microenvironment members, such as immune inflammatory cells and endothelial cells [163], should be investigated.

In breast or colon cancer, evident experimental studies have confirmed a correlation between the disruption of the melatonin rhythm and cancer [164, 165]. Although the epidemiologic surveys suggest that men who worked at night at anytime have a higher risk for lung cancer, with excesses apparent across all main histologic subtypes [50], additional evidence for this from experimental studies is urgently needed. While the anticancer mechanisms of melatonin in breast cancer has been extensively explored [87, 97, 166], the anti-lung cancer actions of melatonin have been generally overlooked.

## CONCLUDING REMARKS

Melatonin may be a potential anticancer drug in the treatment of NSCLC as well as other cancer types. Since melatonin treatment enhances the efficacy and reduces the side-effects of radio- or chemotherapies, and its exogenous supplementation may allow for the use of larger amounts of conventional treatments thereby exaggerating their effectiveness as anticancer treatments. Furthermore, melatonin, *via* its anti-proliferative, pro-apoptotic, anti-metastatic, and immunostimulatory actions, should be given more consideration as an effective oncostatic agent. Inasmuch as melatonin has low toxicity and highly favorable compatibility, further clinical trials which include melatonin and the clarification of additional molecular processes of melatonin's oncostatic effects will help to facilitate better applications of melatonin in the area of NSCLC treatment.
